# Non-local means based Rician noise filtering for diffusion tensor and kurtosis imaging in human brain and spinal cord

**DOI:** 10.1186/s12880-021-00549-9

**Published:** 2021-01-30

**Authors:** Zhongping Zhang, Dhanashree Vernekar, Wenshu Qian, Mina Kim

**Affiliations:** 1 Department of Diagnostic Radiology, The University of Hong Kong, Hong Kong SAR, China; 2 Department of Neuroinflammation, Faculty of Brain Sciences, UCL Queen Square Institute of Neurology, London, UK; 3 Present Address: Philips Healthcare, Shanghai, China; 4 Present Address: Laboratory of Clinical Investigation, National Institute on Aging, NIH, Baltimore, USA

**Keywords:** Rician noise filter, Diffusion tensor imaging, Diffusion kurtosis imaging, Human brain, Human cervical spinal cord

## Abstract

**Background:**

To investigate the effect of using a Rician nonlocal means (NLM) filter on quantification of diffusion tensor (DT)- and diffusion kurtosis (DK)-derived metrics in various anatomical regions of the human brain and the spinal cord, when combined with a constrained linear least squares (CLLS) approach.

**Methods:**

Prospective brain data from 9 healthy subjects and retrospective spinal cord data from 5 healthy subjects from a 3 T MRI scanner were included in the study. Prior to tensor estimation, registered diffusion weighted images were denoised by an optimized blockwise NLM filter with CLLS. Mean kurtosis (MK), radial kurtosis (RK), axial kurtosis (AK), mean diffusivity (MD), radial diffusivity (RD), axial diffusivity (AD) and fractional anisotropy (FA), were determined in anatomical structures of the brain and the spinal cord. DTI and DKI metrics, signal-to-noise ratio (SNR) and Chi-square values were quantified in distinct anatomical regions for all subjects, with and without Rician denoising.

**Results:**

The averaged SNR significantly increased with Rician denoising by a factor of 2 while the averaged Chi-square values significantly decreased up to 61% in the brain and up to 43% in the spinal cord after Rician NLM filtering. In the brain, the mean MK varied from 0.70 (putamen) to 1.27 (internal capsule) while AK and RK varied from 0.58 (corpus callosum) to 0.92 (cingulum) and from 0.70 (putamen) to 1.98 (corpus callosum), respectively. In the spinal cord, FA varied from 0.78 in lateral column to 0.81 in dorsal column while MD varied from 0.91 × 10^−3^ mm^2^/s (lateral) to 0.93 × 10^−3^ mm^2^/s (dorsal). RD varied from 0.34 × 10^−3^ mm^2^/s (dorsal) to 0.38 × 10^−3^ mm^2^/s (lateral) and AD varied from 1.96 × 10^−3^ mm^2^/s (lateral) to 2.11 × 10^−3^ mm^2^/s (dorsal).

**Conclusions:**

Our results show a Rician denoising NLM filter incorporated with CLLS significantly increases SNR and reduces estimation errors of DT- and KT-derived metrics, providing the reliable metrics estimation with adequate SNR levels.

## Background

Diffusion magnetic resonance imaging (dMRI) is widely applied imaging modality allowing to characterize the microstructural properties of brain tissues. Among various kinds of dMRI methods, diffusion tensor imaging (DTI) has been the most commonly used [1–11]. While DTI focuses on the study of white matter (WM) structure providing important information about the tissue anisotropy, diffusion kurtosis imaging (DKI), an extension of DTI provides complementary information especially for analysis of microstructure in gray matter (GM) by probing non-Gaussian diffusion properties [12–16]. In recent years, the interest for DKI has been continuously growing and mean kurtosis (MK), a principal metric of the diffusional non-Gaussianity has demonstrated improved sensitivity in measuring developmental [16–18] and pathological [15, 19] changes in neural tissues of both animal and human over conventional DTI. However, one of the challenges in DKI measurement which reduces its practical usage in clinical research is a use of multiple and higher diffusion-weighting (b-values) as compared to a conventional DTI. This results in a low signal-to-noise ratio (SNR) of which the effect plays a large role in yielding the erroneous tensor estimation due to the bias induced by Rician nature of random noise [20, 21]. The low SNR can also affect the accuracy of diffusion tensor properties such as trace and fractional anisotropy (FA) [11, 22, 23]. Especially, the application of DTI in the spinal cord suffers from the low SNR [24, 25] in addition to other challenges such as the small size of the cord, physiological motion, local field inhomogeneity and susceptibility artefacts [26–29].

To date, various denoising methods have been developed to improve the quality of DW images, such as the Gaussian filter [30, 31], anisotropic diffusion filter [32–34], linear minimum mean squared error filter [21], random matrix theory [35], multi-shell position-orientation-adaptive smoothing [36] and nonlocal means (NLM) filter [37, 38]. In particular, NLM filter has been suggested to significantly improve MR data quality by reducing Rician noise [20, 39–41] and implemented to DTI [42–44] and DKI [45] in the human brain. Moreover, NLM filter has shown to provide efficiency of noise removal while the fine structures and details of images are well preserved [40, 46–51]. Despite considerable research effort in identifying the best denoising algorithm among the existing algorithms for DKI [45, 46, 52] as well as DTI [21, 37, 45, 48, 53], the standard denoising methodology for DKI post-processing part is still to be established, which makes DKI more difficult to use clinically. In order to translate DKI as a clinical tool, it is required to evaluate the direct impact of correcting such bias on regional DKI values associated with SNR in the healthy brain. A few studies investigated inter-subject variability of DKI metrics in the brain of healthy subjects without including noise bias corrections [54, 55]. To the best of our knowledge, only one study [56] investigated the influence of noise correction by data processing related to inter-subject variability of DKI metrics, however the reported diffusion metrics are limited to mean diffusivity (MD), FA and MK. In DTI of spinal cord, previous studies suggested various correction methods to boost SNR [25, 57–61], however the impact of Rician noise filtering in DTI of the human spinal cord has not been reported yet.

In this study, we evaluate the effect of using the Rician NLM filtering in combination with constrained linear least squares (CLLS), one of the least-squares (LS) fitting algorithms commonly used in DTI and DKI studies [13, 62, 63] of the human brain and spinal cord. In particular, we present here diffusivity and kurtosis measures in different anatomical regions of the healthy human volunteers. Additionally, error measures are quantitatively compared between each DT- and KT-metric with and without implementing a Rician NLM filter (CLLS-R and CLLS, respectively).

## Methods

### Subjects

For brain scans, 9 healthy volunteers (5 male, 4 females; mean age = 24; ± standard deviation = 2 years) were recruited through local advertisements. The exclusion criteria for healthy controls were subjects who suffer from any neurological/psychological conditions and any physical disabilities. All participants were studied after signed, informed consent. The study was approved by the local Institutional Review Board. Additionally, the previously obtained spinal cord data including five healthy volunteers (1 male, 4 female; mean age = 32 ± 7 years) [64] were retrospectively used in the present study for Rician denoising filtering.

### Data acquisition

All MRI scans were performed on a Philips 3 T MRI Achieva scanner (Philips Healthcare, Best, The Netherlands). Brain scans were carried out with a body coil excitation and an 8-channel SENSE head coil for reception. For DKI data, four averaged minimally weighted (b_0_) and 32 non-collinear diffusion-encoding directions with two b-values (1000 and 2000 s/mm^2^) were acquired using single-shot EPI sequence as similar to previous studies [65, 66]. The imaging parameters were: TR/TE = 2000/69 ms, FOV = 224 × 224 mm^2^, acquisition matrix = 88 × 88, reconstructed resolution = 2 × 2 mm^2^, fold-over direction = AP, EPI factor = 47, 44 axial slices with 3 mm thickness and no inter-slice gap to cover the whole brain, SENSE factor = 2, 3/4 partial Fourier encoding, 2 averages and total scan time = 19 min. 38 s. The number of averages for DWIs was optimized to sustain the reasonable SNR while compromising scan time and six sequential observations of the DWIs with 1 average were obtained in the additional scans for computing the SNR. For anatomical reference, three-dimensional T_1_-weighted images were also acquired using 3D-MPRAGE sequence with the following parameters: TR/TE = 7.0/3.2 ms, TI = 800–818 ms, FOV = 224 × 224 × 167 mm^3^, acquisition matrix = 224 × 224, reconstruction resolution = 1 × 1 mm^2^, 167 sagittal slices with 1 mm thickness and no inter-slice gap to cover the whole brain, 1 average and total scan time = 10 min. 41 s. Acquisition parameters for spinal cord DTI data can be found from the previous study [64] which included four averaged minimally weighted (b_0_) and 15 diffusion-weighted volumes (b-value = 500 s/mm^2^) using single-shot echo-planar-imaging (EPI) sequence. Two imaging stacks in axial plane covered upper (C1 to C3) and lower (C4 to C6) portions of the cervical spinal cord (slice thickness = 2.5 and 5 mm, respectively).

### Data postprocessing

**Fig. 1:**
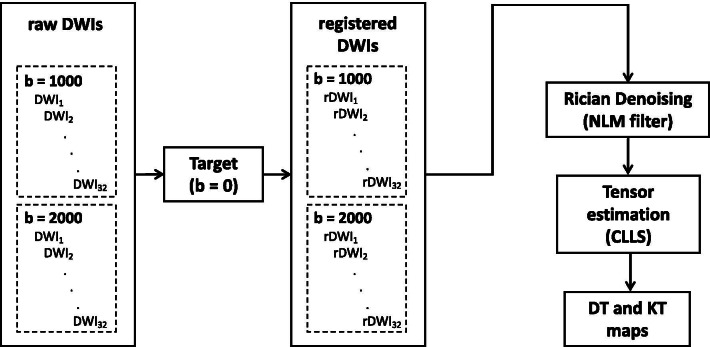
Data processing pipeline

Data were analysed using a custom-written program in Matlab (Mathworks, Natick, MA) and the DKE software (https://www.nitrc.org/projects/dke/) [52]. Detailed data postprocessing procedures for DKI are illustrated in Fig. 1. All DWIs were co-registered to the initial b_0_ image using a 6-degree-of-freedom, rigid body transformation procedure supplied in automated image registration (AIR) [67] in order to reduce the impact of motion artefact and eddy current distortion. Each gradient direction was revised with respect to transformation matrix after registration. Prior to tensor estimation, registered DWIs were denoised by an optimized blockwise NLM filter consisting of a blockwise implementation with automatic tuning of the smoothing parameter and block selection as described elsewhere [39, 40]. The numerical value for SNR in regions of interest (ROIs) for baseline image (b = 0) was computed by the signal statistics in a difference image as described in Farrell et al. [68]. DT and KT were estimated using CLLS algorithm suggested by Tabesh et al. [52] based on the definition of all DT- and KT-metrics [10, 14, 52].

### ROI-based measurements

**Fig. 2:**
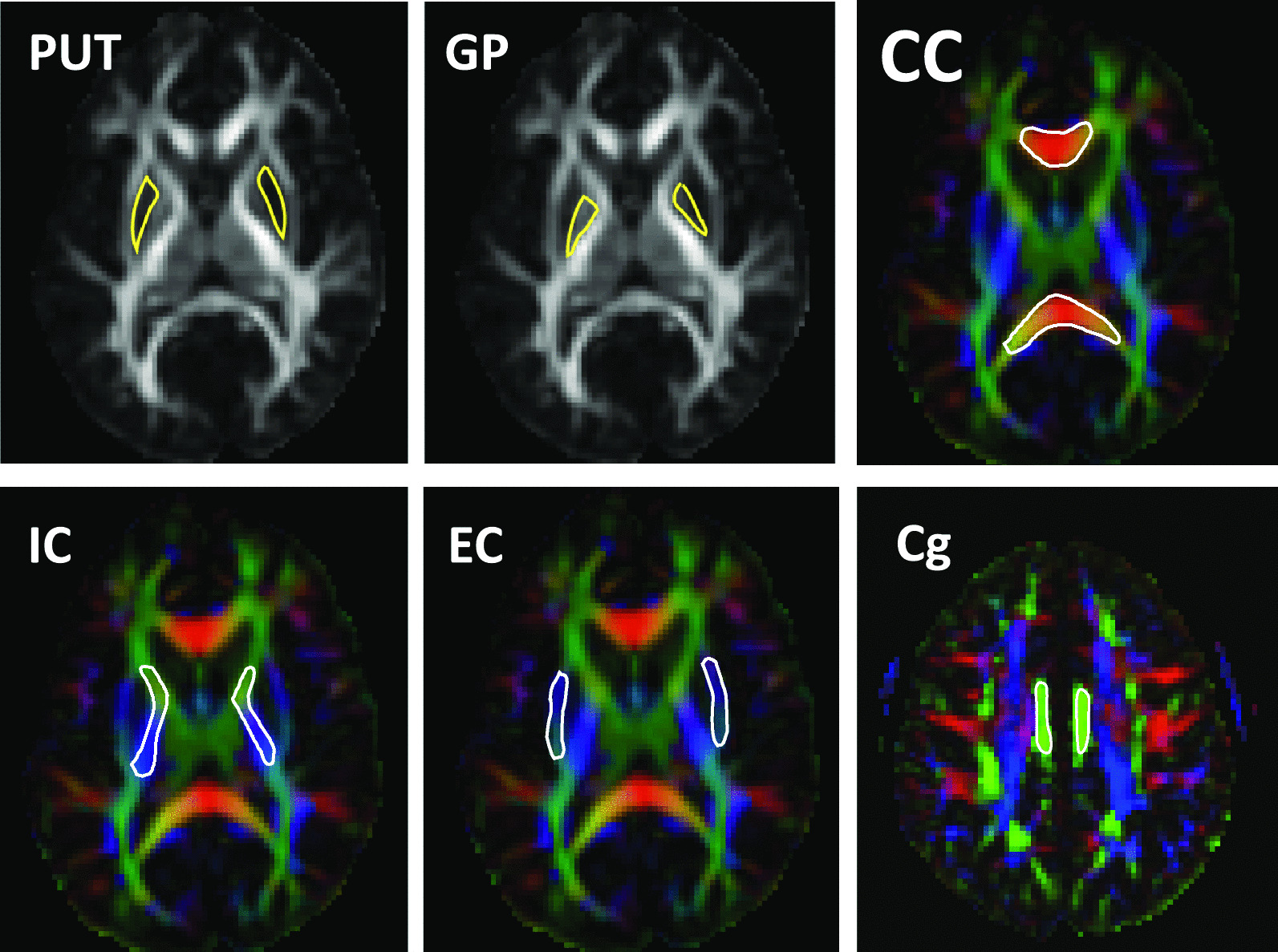
Location of ROIs on the FA and color-coded maps of the brain. *Abbreviations* PUT, putamen; GP, globus pallidus; CC, corpus callosum; IC, internal capsule; EC, external capsule; Cg, cingulum

For brain images, 6 ROIs including putamen (PUT; averaged area size = 985 mm^2^), globus pallidus (GP; averaged area size = 525 mm^2^), corpus callosum (CC; averaged area size = 2497 mm^2^), internal capsule (IC; averaged area size = 1643 mm^2^), external capsule (EC; averaged area size = 1241 mm^2^) and cingulum (Cg; averaged area size = 678 mm^2^) were manually drawn in a FA map and a color-coded map using ImageJ (National Institutes of Health, Bethesda, MD) by a trained rater (Z.Z.) referencing to the standard human brain atlas (Fig. 2) [69]. All 6 ROIs were then transferred to the rest of the DT- and KT-derived maps in order to compute the average and error measures within the ROIs for each subject. Chi-square value (*χ*^2^) was calculated by computing the difference between the observed and estimated signals to estimate the goodness of fitting. Later, another trained rater (W.Q.) independently placed ROIs on all datasets to ensure the indices were reliable across raters.

For spinal cord images, 6 slices matching C1 to C6 levels of the spinal cord in a FA map per each subject, were selected around mid-level of two intervertebral discs to minimize susceptibility artefact typically caused near the disc-tissue boundaries. In each chosen slice, 3 ROIs were manually placed in the lateral left and right, and dorsal columns (Fig. 4a), a couple of pixels away from the boundaries of GM/WM and WM/cerebrospinal fluid (CSF) to avoid partial volume effect using ImageJ (National Institutes of Health, Bethesda, MD, USA). The area size of 1.563 mm^2^ for each ROI was consistent across all subjects. These ROIs were then transferred to the rest of the DTI-derived maps including mean FA, MD, radial diffusivity (RD) and axial diffusivity (AD), in order to compute the average of DTI-indices within the ROIs for each subject. *χ*^2^ was calculated in a voxel-by-voxel basis to evaluate the goodness of denoising performance. SNR and DTI-derived metrics were recalculated without the prior-denoising process and using the same ROIs that were previously defined for comparison.

### Statistical analysis

To evaluate the inter-rater reliability, the intraclass correlation coefficient (ICC) was computed to assess reliability of mean values measured by two raters for each ROI as described by Shrout and Fleiss [70]. Averaged SNR values of b_0_ images and ROI-based diffusion and kurtosis metrics were compared between CLLS and CLLS-R by paired Mann–Whitney U test. All statistical analyses were performed using commercially available software (SPSS, Chicago, IL, USA). *P*-values of 0.05 were considered to be significant.

## Results

In a single b_0_ image of the brain, SNR ranged from 10.85 (GP) to 20.50 (EC) with CLLS (without Rician NLM filtering) and from 25.25 (CC) to 74.38 (EC) with CLLS-R (with Rician NLM filtering) (Table 1). SNR significantly increased in all 6 ROIs using CLLS-R (244%, 177%, 62%, 162%, 263% and 125% for PUT, GP, CC, IC, EC and Cg, respectively with *p* < 0.05 for all ROIs) as compared to CLLS. In upper spinal cord levels C1–C3 (2.5 mm slice thickness), SNR ranged from 9.77 (Lateral) to 10.66 (Dorsal) with CLLS and from 16.32 (Lateral) to 21.97 (Dorsal) with CLLS-R (Table 2). In lower spinal cord levels C4–C6 (5 mm slice thickness), SNR ranged from 23.02 (Dorsal) to 23.73 (Lateral) with CLLS and from 45.21 (Dorsal) to 49.23 (Lateral) with CLLS-R (Table 2). In C1–C3, mean SNR significantly increased by 67.09% and 106.11% for lateral and dorsal respectively (*p* < 0.001 for both columns). Similarly, significant increase of mean SNR by 107.50% and 96.38% was observed for lateral and dorsal respectively in C4–C6 (*p* < 0.001 for both columns).

**Table 1:** Comparison of averaged SNR ± standard deviation of b_0_ images in the brain of healthy volunteers (n = 9) measured using CLLS and CLLS-R. Paired Mann–Whitney U test was used in the comparison between CLLS and CLLS-R

	CLLS	CLLS-R	*p*
PUT	17.65 ± 0.73	60.75 ± 6.27	< 0.05
GP	10.85 ± 0.62	30.02 ± 4.81	< 0.05
CC	15.61 ± 1.19	25.25 ± 2.54	< 0.05
IC	15.78 ± 0.28	41.30 ± 3.88	< 0.05
EC	20.50 ± 1.14	74.38 ± 4.39	< 0.05
Cg	19.56 ± 2.23	44.04 ± 4.42	< 0.05

SNR, signal-to-noise ratio; PUT, putamen; GP, globus pallidus; CC, corpus callosum; IC, internal capsule; EC, external capsule; Cg, cingulum

**Table 2:** Mean ± standard deviation of averaged SNR of b_0_ images in 5 healthy volunteers in lateral & dorsal columns before and after denoising

	Up (C1-C3)			Low (C4-C6)		
	CLLS	CLLS-R	*p*	CLLS	CLLS-R	*p*
Lateral	9.77 ± 0.47	16.32 ± 3.72	< 0.001	23.73 ± 1.24	49.23 ± 5.33	< 0.001
Dorsal	10.66 ± 0.71	21.97 ± 3.77	< 0.001	23.02 ± 0.56	45.21 ± 7.03	< 0.001

The right and left lateral columns have been averaged together for this analysis. Paired Mann–Whitney U test was used in the comparison between CLLS and CLLS-R

**Fig. 3:**
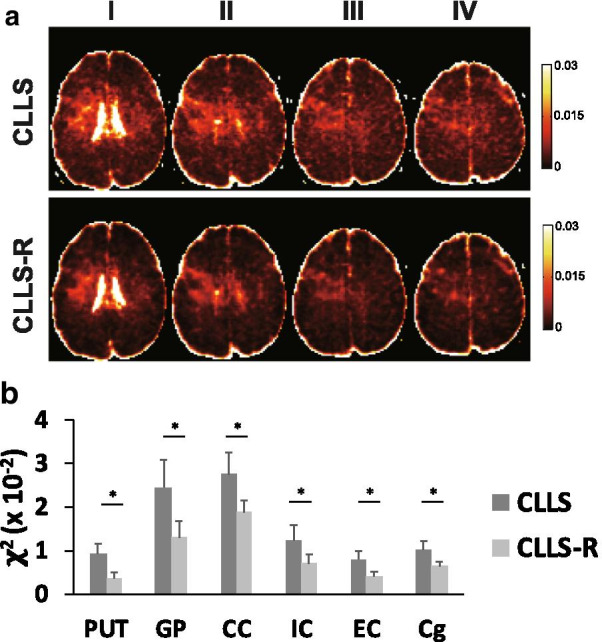
**a** Representative 4 neighbouring axial slices of *χ*^2^ maps in a healthy subject (25 years old, male) using CLLS and CLLS-R. **b** Group differences of the *χ*^2^ measurement at each brain region between before- (CLLS) and after-Rician denoising (CLLS-R). Significant differences between groups are indicated by *p < 0.05. *Abbreviations* PUT, putamen; GP, globus pallidus; CC, corpus callosum; IC, internal capsule; EC, external capsule; Cg, cingulum

**Fig. 4:**
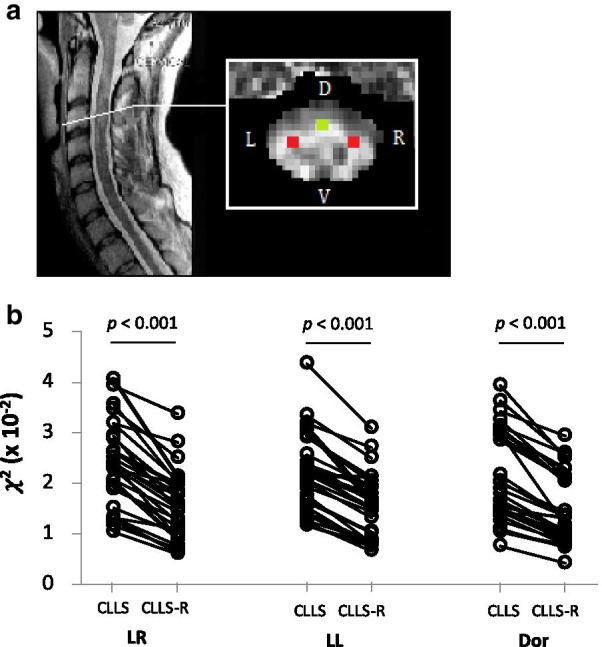
**a** Location of anatomically defined ROIs on the FA map of the spinal cord. ROIs were selected in the ventro-lateral (red) and dorsal (green) column of the cervical spinal cord, to include most descending and ascending tracts, respectively. **b** The before-and-after plots for mean *χ*^2^ values at each ROI from 6 spinal cord levels (C1-C6) of all 5 healthy volunteers (30 pairs). All pairs show significant differences between before- (CLLS) and after-Rician denoising (CLLS-R) (*p* < 0.001). *Abbreviations* LR, lateral right; LL, lateral left; Dor, dorsal

**Fig. 5:**
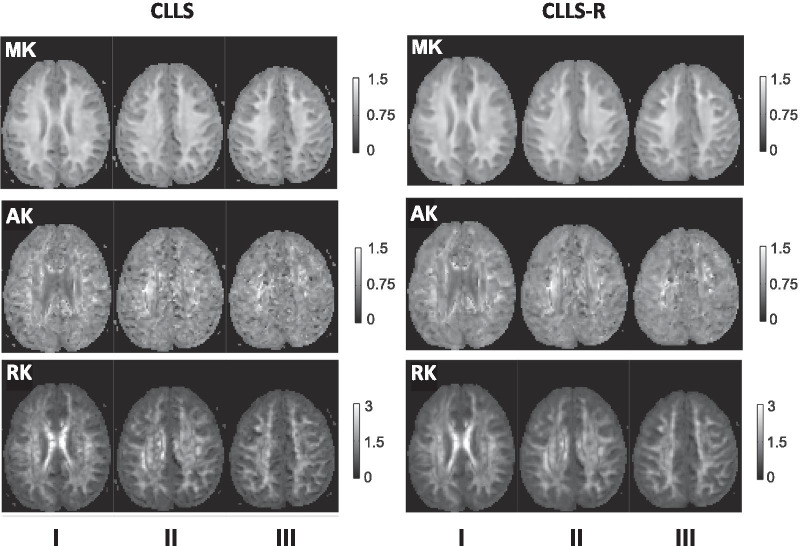
Representative 3 axial slices of mean kurtosis (MK), axial kurtosis (AK) and radial kurtosis (RK) maps calculated using CLLS and CLLS-R. Scale bars represent dimensionless range of KT-derived metrics

The *χ*^2^ maps in the brain of a representative subject (25, male) (Fig. 3a) and group differences (Fig. 3b) demonstrate that *χ*^2^ values estimated by CLLS-R are decreased in deep GM, WM and overall cortical GM as compared to those by CLLS. Group comparison averaged across 9 subjects at each ROI shows that mean *χ*^2^ values significantly decreased in all 6 regions of brain using CLLS-R as compared to CLLS (32—61% with *p* < 0.05). Similarly, the averaged *χ*^2^ values of the spinal cord over 5 healthy subjects significantly decreased by maximum 43% with Rician denoising as compared those without Rician denoising in all 3 columns throughout C1–C6 (*p* < 0.001) (Fig. 4B). In the representative DT- and KT-derived brain maps, KT-derived metrics (MK, axial kurtosis (AK) and radial kurtosis (RK)) show visual difference between CLLS and CLLS-R (Fig. 5). Kurtosis maps (AK and RK) using CLLS-R illustrate significant decrease in a number of erroneous pixels as compared to those using CLLS while moderate improvement is noticeable in MK maps (Fig. 5).

All the KT-derived metrics (MK, RK, and AK), together with the DT-derived metrics (FA, MD, AD, and RD) by CLLS-R averaged across all subjects at each ROI are listed in Table 3. In the brain, MK varied from 0.70 to 1.27 with the lowest MK value obtained in PUT and highest in IC while RK varied from 0.70 (PUT) to 1.98 (CC). AK varied from 0.58 in the CC to 0.92 in Cg. FA varied from 0.12 to 0.65, with the lowest FA value obtained in the PUT and highest in CC while MD varied from 0.75 × 10^−3^ mm^2^/s (PUT) to 1.04 × 10^−3^ mm^2^/s (CC). RD varied from 0.50 × 10^−3^ mm^2^/s in IC to 0.71 × 10^−3^ mm^2^/s in PUT and AD varied from 0.85 × 10^−3^ mm^2^/s in PUT to 1.95 × 10^−3^ mm^2^/s in CC. ICC of DT- and KT-indices measured in various ROIs by two raters ranged from 0.744 to 0.999 (Table 4). In the spinal cord, FA varied from 0.78 in lateral column to 0.81 in dorsal column while MD varied from 0.91 × 10^−3^ mm^2^/s (lateral) to 0.93 × 10^−3^ mm^2^/s (dorsal). RD varied from 0.34 × 10^−3^ mm^2^/s (dorsal) to 0.38 × 10^−3^ mm^2^/s (lateral) and AD varied from 1.96 × 10^−3^ mm^2^/s (lateral) to 2.11 × 10^−3^ mm^2^/s (dorsal). DT- and KT-derived metrics estimated using CLLS-R were significantly different from those by CLLS in most of examined anatomical regions (Additional File 1: Supporting Information Table S1). The mean FA values using CLLS-R were significantly decreased in all 6 regions (3–11% with *p* < 0.05) compared to CLLS. Similar results were found for AD with exception of the PUT, CC and Cg. Meanwhile MD, RD, RK and MK values were significantly increased with exception of the PUT, IC and EC for MD and PUT for RD, CC and Cg for RK and PUT for MK. Additionally, the ROI-based mean *χ*^2^ values showed that CLLS-R significantly reduced *χ*^2^ values over all ROIs compared to CLLS (32—61% with *p* < 0.05).

**Table 3:** ROI-based mean ± standard deviation of DT- and KT-indices averaged across all subjects at each ROI in the brain and the spinal cord using CLLS-R

	MK	RK	AK	MD (10^–3^ mm^2^/s)	FA	RD (10^–3^ mm^2^/s)	AD (10^–3^ mm^2^/s)
*Brain*							
PUT	0.70 ± 0.04	0.70 ± 0.04	0.72 ± 0.04	0.75 ± 0.03	0.12 ± 0.02	0.71 ± 0.02	0.85 ± 0.04
GP	1.03 ± 0.06	1.13 ± 0.09	0.89 ± 0.08	0.78 ± 0.04	0.26 ± 0.02	0.68 ± 0.03	0.99 ± 0.06
CC	1.09 ± 0.04	1.98 ± 0.10	0.58 ± 0.02	1.04 ± 0.05	0.65 ± 0.02	0.58 ± 0.05	1.95 ± 0.07
IC	1.27 ± 0.01	1.94 ± 0.05	0.71 ± 0.03	0.82 ± 0.02	0.59 ± 0.02	0.50 ± 0.02	1.44 ± 0.05
EC	0.88 ± 0.04	1.14 ± 0.06	0.72 ± 0.03	0.82 ± 0.02	0.32 ± 0.03	0.68 ± 0.02	1.11 ± 0.05
Cg	0.99 ± 0.03	1.51 ± 0.16	0.92 ± 0.02	0.86 ± 0.03	0.42 ± 0.04	0.65 ± 0.04	1.29 ± 0.07
*Spinal Cord*							
LR	−	−	−	0.91 ± 0.05	0.78 ± 0.09	0.38 ± 0.06	1.96 ± 0.05
LL	–	−	−	0.93 ± 0.07	0.79 ± 0.08	0.38 ± 0.05	2.01 ± 0.09
Dor	–	−	−	0.93 ± 0.04	0.81 ± 0.07	0.34 ± 0.03	2.11 ± 0.08

PUT, putamen; GP, globus pallidus; CC, corpus callosum; IC, internal capsule; EC, external capsule; Cg, cingulum; LR, lateral right; LL, lateral left; Dor, dorsal

**Table 4:** Intraclass correlation coefficient of DT- and KT-indices measured in various ROIs by two raters^a^

	MK	RK	AK	MD	FA	RD	AD
PUT	0.911	0.749	0.959	0.971	0.856	0.985	0.947
GP	0.893	0.914	0.893	0.956	0.768	0.930	0.905
GM^b^	0.992	0.991	0.968	0.967	0.988	0.963	0.980
CC	0.949	0.974	0.962	0.896	0.906	0.906	0.876
IC	0.857	0.910	0.792	0.974	0.744	0.892	0.884
EC	0.910	0.823	0.968	0.987	0.926	0.911	0.978
Cg	0.938	0.971	0.834	0.995	0.982	0.992	0.977
WM^c^	0.997	0.995	0.991	0.992	0.996	0.985	0.998
All ROIs	0.996	0.997	0.988	0.994	0.998	0.990	0.999

PUT, putamen; GP, globus pallidus; CC, corpus callosum; IC, internal capsule; EC, external capsule; Cg, cingulum; GM, gray matter; WM, white matter

^a^The *p* value is < 0.001 for all metrics in all ROIs

^b^GM includes PUT and GP

^c^WM includes CC, IC, EC and Cg

## DISCUSSION

Despite great deal of effort in comparing various denoising algorithms for DKI in previous studies [45, 46, 52] and inter-subject variability of DKI metrics in brain of healthy subjects [54, 55], the direct impact of Rician noise correction on DKI data has not been sufficiently studied, especially related to improvement on SNR and error measures in various anatomical regions. In this study, we evaluate the influence of the Rician NLM filter in combination with CLLS algorithm on the brain DKI and the spinal cord DTI data. Rician NLM filter in combination with CLLS algorithm has the potential to be useful in clinical research because it is easy and efficient to implement. To the best of our knowledge, this is the first study to assess the influence of combining the NLM filter with CLLS in DTI of the spinal cord and DKI in the brain and to report ROI-based changes in SNR and the associated error estimates. To compare diffusional measures using different processing methods (CLLS and CLLS-R), we obtained whole brain DKI data from 9 healthy volunteers and compared mean DT- and KT-derived indices using CLLS and CLLS-R. Rician NLM filter was also applied to the previously acquired spinal cord data retrospectively [64].

### Measurement of SNR

SNR assessment is important for reliable quantification of diffusional metrics [71]. When SNR is low, Rician noise does not only cause random fluctuations but also a signal dependent bias to the data, which may lead to difficulty in postprocessing such as tensor calculation. However, SNR levels are not routinely reported although previous reports suggest that low SNR causes a bias in FA which may vary with numerous other technical factors such as the region of brain being studied, field strength, hardware and software [68, 72, 73]. As a first step to evaluate the influence of Rician denoising, we have measured SNR in various anatomical areas of the brain and the spinal cord with and without Rician denoising. Our results show that Rician NLM filtering yields the significant increase of SNR in the brain DKI and the spinal cord DTI data (Table 1 and 2). The ROI-based SNR values with noise correction in our study are consistent with the previous study by Seo et al. [74] which has reported SNR thresholds 20 in the CC and 70 in the PUT for bias-free estimation of tensor metrics. It should be noted that those SNR values from the literatures were obtained from different acquisition protocols. Thus, when comparing those values with literature values, care should be taken to ensure the similarity of protocol chosen for comparison. For instance, the eddy current and off-resonance effects in a DWI sequence may substantially vary with b-value and diffusion gradient direction. Additionally, the spatial noise distribution can be varied by coil geometry, phase-encoding direction and acceleration factor of parallel imaging [38, 75, 76] which also need to be taken into account for. In our study, it is also observed that the degree of SNR improvement relates to the underlying structures. For instance, the CC has the smallest SNR increase among other regions, which might be attributed to the previous findings that the noise within accelerated images is nonhomogeneous with higher signal peripherally and noise centrally when parallel imaging is used [77–79]. As the levels and spatial distributions of noise are not an equal across the DW image, it is expected that various anatomical areas with different SNR requirements have diverse range of SNR increase rate.

While denoising DTI with low SNR addressing the strong influence of Rician bias in the brain has been well presented by different studies [48, 80, 81], Rician NLM denoising has not been established in the spinal cord DTI where most of previous studies have focused on data acquisition or motion correction methods to improve SNR [25, 27, 58, 82]. The mean SNR values without Rician denoising in C1-C3 in our study are within a range of values from a multi-centre study reported by Samson et al. (6.74 – 10.9) [27]. However, our mean SNR values significantly increased in both lateral and dorsal areas of the spinal cord levels C1-C6 after a Rician denoising (Table 2). Our results indicate that a simple noise correction method in the spinal cord such as Rician denoising being used in our study increases SNR (Table 2) and thereby can reduce error estimates (Fig. 4b). This is not surprising given the improved quantification of dMRI in the brain, however worth reporting for the growing need of the spinal cord DTI in the clinical research despite more intrinsic challenges in the spinal cord as compared to the brain.

### Estimation errors

Significantly reduced number of erroneous pixels (black holes) was observed in AK, RK and MK using CLLS-R as compared to CLLS in the brain (Fig. 5). In particular, clear difference is observed in cortical GM, where the voxel values with the erroneously estimated tensor are lower using CLLS as compared to CLLS-R (Fig. 3a). Note that voxels with extreme (negative or zero tensor) values were excluded from the computation. Even after all of extremely erroneous voxels were removed, the ROI-based mean *χ*^2^ values significantly decreased over all ROIs when estimated using CLLS-R compared to CLLS in all examined anatomical regions (Fig. 3b), suggesting the impact of Rician denoising step towards KT estimation in the brain. Additionally, the mean *χ*^2^ values in the spinal cord show that Rician NLM filtering significantly reduced error estimates in both lateral and dorsal columns (Fig. 4b). Reduced *χ*^2^ values both in the brain and the spinal cord, imply that accuracy of tensor estimation is significantly improved with Rician NLM filtering (Figs. 3b and 4b).

### Regional values of DT- and KT-derived metrics

Currently, a few reports in the literature are available for comparison with our results [54–56, 83, 84]. Table 5 provides an overview of those works in the literature that include values of parameter estimates. While CLLS estimation led to overestimated DT-derived metrics, especially in FA (Additional File 1: Supporting Information Table S1), which is a common problem in human DTI studies [68, 85, 90], the DT- and KT-derived metrics using CLLS-R are consistent with some of the previously reported values (Table 3). Nevertheless, it is observed that the discrepancy in diffusional metrics values exists amongst various studies in the literature. There are a few factors that may explain discrepancy between studies, from data acquisition to postprocessing perspective. In DTI, it has been well known that low SNR causes a bias in DT-derived metrics, leading to overestimation of FA [68, 72, 73]. When SNR is low, Rician noise does not only cause random fluctuations but also a signal dependent bias to the data, which may lead to difficulty in postprocessing such as tensor calculation. Therefore bias-free measurements require adequate SNR [85] and DTI studies often report SNR values to assure the reliability of the estimated metrics. However, SNR levels are not routinely reported in DKI, resulting in the challenges of comparing results between studies. Therefore it is desirable to ensure the DT- and KT-derived indices are estimated with adequate SNR levels before the comparison between studies. Our results show that the mean MK varied from 0.70 (PUT) to 1.27 (IC) while AK and RK varied from 0.58 (CC) to 0.92 (Cg) and from 0.70 (PUT) to 1.98 (CC), respectively, with a range of SNR levels (from 25.25 in CC to 60.75 in PUT) in various anatomical structures of the brain. Additionally, inter-subject differences substantially contribute the within-group variability [86], and may partially explain discrepancy between studies. In particular, various choice of ROIs among studies (i.e. selection of structure from only contiguous voxels with the highest values to the entire structure) hampers comparisons between studies. For instance, the difference of FA in the genu of the CC between 0.44 in [55] and 0.80 in [54], may be largely due to the ROI used for measurement. Therefore it is important to ensure the DT- and KT-derived indices are reliable across raters within-group, as ROI-based measurement often required rater decisions which may have impacted ROI placement between observers. Our results show that ICC values are near 1 for various ROIs over all metrics, indicating high reliability of ROI-based measurement performed in the brain. It should be also noted that regional variability of DTI values between publications may relate to age differences within-group aside from selection of ROIs, acquisition parameters and SNR. Considerable inter-subject variability of DTI parameters has been shown in previous studies that mostly reported the age dependence of DTI metrics [87–89]. Our subjects were young adults (mean age 24 ± 2) and this may contribute on variability of diffusion metrics as compared to those in the literature.

**Table 5:** Regional values in the healthy brain from the literature

Region	References	Number of directions (DIR), b-values (ms/μm^2^)	Voxel size (mm^3^)	MK	RK	MD (mm^2^/s)	FA	RD (mm^2^/s)
PUT	[54]	15 DIR, 5 b-values (0, 500, 1000, 2500, 2750)	2 × 2 × 2	0.67 ± 0.08	0.61 ± 0.08	0.79 ± 0.03	0.15 ± 0.02	0.73 ± 0.03
PUT	[55]	50 DIR, 3 b-values (0, 1000, 2000)	1.9 × 1.9 × 5	0.77 ± 0.01	0.85 ± 0.01	1.72 ± 0.01	0.16 ± 0.01	1.57 ± 0.03
GP	[54]	15 DIR, 5 b-values (0, 500, 1000, 2500, 2750)	2 × 2 × 2	1.06 ± 0.08	1.05 ± 0.10	0.86 ± 0.08	0.27 ± 0.04	0.74 ± 0.06
GP (Left) GP (Right)	[56]	60 DIR, 3 b-values (0, 1000, 2800)	2.2 × 2.2 × 2.2	1.76 ± 0.15 1.85 ± 0.17	–	–	–	–
CC (genu)	[54]	15 DIR, 5 b-values (0, 500, 1000, 2500, 2750)	2 × 2 × 2	1.06 ± 0.11	2.07 ± 0.45	0.93 ± 0.06	0.80 ± 0.04	0.36 ± 0.07
CC (genu)	[55]	50 DIR, 3 b-values (0, 1000, 2000)	1.9 × 1.9 × 5	0.90 ± 0.05	0.90 ± 0.07	1.82 ± 0.08	0.70 ± 0.05	1.04 ± 0.07
CC (genu)	[83]	64 DIR, 3 b-values (0, 1000, 2000)	2.5 × 2.5 × 2.5	0.94 ± 0.07	–	1.38 ± 0.12	0.44 ± 0.04	–
CC (splenium)	[54]	15 DIR, 5 b-values (0, 500, 1000, 2500, 2750)	2 × 2 × 2	1.32 ± 0.09	2.72 ± 0.41	0.89 ± 0.09	0.83 ± 0.03	0.31 ± 0.07
CC (splenium)	[55]	50 DIR, 3 b-values (0, 1000, 2000)	1.9 × 1.9 × 5	1.07 ± 0.08	1.05 ± 0.07	1.70 ± 0.06	0.76 ± 0.04	0.87 ± 0.03
CC (splenium)	[83]	64 DIR, 3 b-values (0, 1000, 2000)	2.5 × 2.5 × 2.5	1.14 ± 0.09	–	1.17 ± 0.10	0.54 ± 0.05	–
IC	[84]	15 DIR, 6 b-values (0, 500, 1000, 1500, 2000, 2500)	2 × 2 × 2	1.05 ± 0.08	0.84 ± 0.03	–	–	–
IC (Left) IC (Right)	[56]	60 DIR, 3 b-values (0, 1000, 2800)	2.2 × 2.2 × 2.2	1.45 ± 0.06 1.49 ± 0.07	–	–	–	–
ALIC	[54]	15 DIR, 5 b-values (0, 500, 1000, 2500, 2750)	2 × 2 × 2	1.04 ± 0.10	1.60 ± 0.28	0.87 ± 0.05	0.60 ± 0.04	0.53 ± 0.05
PLIC	[54]	15 DIR, 5 b-values (0, 500, 1000, 2500, 2750)	2 × 2 × 2	1.23 ± 0.09	2.04 ± 0.23	0.89 ± 0.09	0.71 ± 0.04	0.45 ± 0.07
EC	[54]	15 DIR, 5 b-values (0, 500, 1000, 2500, 2750)	2 × 2 × 2	0.81 ± 0.05	1.02 ± 0.09	0.90 ± 0.05	0.41 ± 0.03	0.70 ± 0.04
EC	[55]	50 DIR, 3 b-values (0, 1000, 2000)	1.9 × 1.9 × 5	0.85 ± 0.01	0.93 ± 0.05	1.73 ± 0.19	0.38 ± 0.03	1.28 ± 0.03
Cg	[54]	15 DIR, 5 b-values (0, 500, 1000, 2500, 2750)	2 × 2 × 2	1.07 ± 0.07	1.85 ± 0.26	0.86 ± 0.07	0.66 ± 0.06	0.48 ± 0.08
Cg	[55]	50 DIR, 3 b-values (0, 1000, 2000)	1.9 × 1.9 × 5	0.94 ± 0.03	0.96 ± 0.07	1.64 ± 0.05	0.55 ± 0.05	1.08 ± 0.05

PUT, putamen; GP, globus pallidus; CC, corpus callosum; IC, internal capsule; ALIC, anterior limb of IC; PLIC, posterior limb of IC; EC, external capsule; Cg, cingulum

In the spinal cord, there are numerous reports in the literature available for comparison with our results. In order to ensure the similarity of the acquisition sequences and parameters, here we focus on comparing our DT-derived values with those by Qian et al. [64] (a study that our raw data were originally obtained) and Samson et al. [27] (a multi-centre study with the compatible acquisition protocols as ours). Our FA values with Rician denoising are found to be significantly lower than those previous reported by Qian et al. (0.81–0.84; *p* < 0.05) while our MD, AD and RD values with Rician denoising are not significantly different from those from the same study [64]. This suggests that Rician NLM filtering might reduce overestimation of FA in the spinal cord, which is common observed in DTI of the brain [68, 85, 90], by decreasing estimation error. Our column specific measurements of MD are at the lower end of the previously reported range of 0.93–1.29 mm^2^/ms by Samson et al. [27] while FA measurements are higher than the range measured by the same study (0.59–0.63). Our RD values are lower than the range measured by Samson et al. (0.68–0.84 mm^2^/ms) [27] while AD values are at the higher end of the previously reported range of 1.43–2.22 mm^2^/ms by the same study. Overall, DTI metrics are likely to be within similar ranges, however difference among protocols and the associated SNR in each study must be taken into account when comparing between those DTI metrics.

### Consideration for clinical applications

It is worth noting that the DKI data acquisition took around 20 min per person, because we obtained whole brain DKI data with 2 averages in order to evaluate quality of different processing methods through a whole brain with reasonable SNR. Our results showed significant decrease of *χ*^2^ values, indicating quality of DKI-derived maps improved through a whole brain within reasonable scan time when CLLS-R estimation was performed. Thus, by reducing the number of slices carefully selected for areas of interest, neurology- or neuroscience-related application studies should be feasible, which would last a clinically acceptable time frame (less than 10 min). Additionally, Rician NLM denoising in combination with CLLS can be readily implemented as it is based on a LSE algorithm available through existing commercial programs. Therefore, practical use of the combined Rician denoising method is widely expected in characterizing microarchitectural integrity of normal and pathological states.

## Conclusions

We have demonstrated that a Rician denoising filter incorporated with CLLS (CLLS-R) significantly increases SNR while reducing estimation errors. Our results suggest that the combined postprocessing method may provide the capability to more reliably quantify tissue properties both in the brain and the spinal cord with adequate SNR levels while it is easy to implement. Future studies are warranted towards investigating clinical and neuroscientific applications using this method.

## Supplementary Information

**Additional file 1.** Supporting Information Table S1 showing comparison for DT- and KT-derived metrics between CLLS and CLLS-R analyses.

## Data Availability

All data are fully available upon reasonable request. The corresponding author should be contacted if someone wants to request the data.

## Abbreviations

dMRI: Diffusion magnetic resonance imaging
DTI: Diffusion tensor imaging
WM: White matter
DKI: Diffusion kurtosis imaging
GM: Gray matter
FA: Fractional anisotropy
MD: Mean diffusivity
RD: Radial diffusivity
AD: Axial diffusivity
MK: Mean kurtosis
RK: Radial kurtosis
AK: Axial kurtosis
SNR: Signal-to-noise ratio
NLM: Nonlocal means
CLLS: Constrained linear least squares
CLLS-R: Constrained linear least squares with Rician denoising
LS: Least-squares
EPI: Echo-planar-imaging
AIR: Automated image registration
ROIs: Regions of interest
PUT: Putamen
GP: Globus pallidus
CC: Corpus callosum
IC: Internal capsule
EC: External capsule
Cg: Cingulum
*χ*^2^: Chi-square
CSF: Cerebrospinal fluid
ICC: Intraclass correlation coefficient
