# Supplementary material for: Non-local means based Rician noise filtering for diffusion tensor and kurtosis imaging in human brain and spinal cord
Source: BMC Med Imaging. 2021 Jan 30;21:16. doi: 10.1186/s12880-021-00549-9 (PMC7847150; doi:10.1186/s12880-021-00549-9)
Supplement: Supplementary file 1 — Additional file 1. Supporting Information Table S1 showing comparison for DT- and KT-derived metrics between CLLS and CLLS-R analyses. [file 12880_2021_549_MOESM1_ESM.docx]

**Supporting Information Table S1.** ROI-based analysis of the diffusion and kurtosis metrics. Each index value was averaged across 6 subjects for various ROIs using CLLS and CLLS-R. The paired Mann-Whitney U test was used to compare averaged indices between CLLS and CLLS-R.

|  | FA | | | MD (x 10^-3^ mm^2^/s) | | |
| --- | --- | --- | --- | --- | --- | --- |
|  | CLLS | CLLS-R | *p* | CLLS | CLLS-R | *p* |
| PUT | 0.12 | 0.12 | <0.05 | 0.75 | 0.75 | 0.48 |
| GP | 0.27 | 0.26 | <0.05 | 0.78 | 0.78 | <0.05 |
| CC | 0.67 | 0.65 | <0.05 | 1.02 | 1.04 | <0.05 |
| IC | 0.61 | 0.59 | <0.05 | 0.82 | 0.82 | <0.05 |
| EC | 0.36 | 0.32 | <0.05 | 0.82 | 0.82 | 0.27 |
| Cg | 0.46 | 0.42 | <0.05 | 0.83 | 0.86 | <0.05 |
|  | AD (x 10^-3^ mm^2^/s) | | | RD (x 10^-3^ mm^2^/s) | | |
|  | CLLS | CLLS-R | *p* | CLLS | CLLS-R | *p* |
| PUT | 0.85 | 0.85 | 0.94 | 0.71 | 0.71 | 0.31 |
| GP | 1.00 | 0.99 | <0.05 | 0.67 | 0.68 | <0.05 |
| CC | 1.94 | 1.95 | <0.05 | 0.55 | 0.58 | <0.05 |
| IC | 1.48 | 1.44 | <0.05 | 0.49 | 0.50 | <0.05 |
| EC | 1.15 | 1.11 | <0.05 | 0.66 | 0.68 | <0.05 |
| Cg | 1.29 | 1.29 | 0.58 | 0.60 | 0.65 | <0.05 |
|  | AK | | | RK | | |
|  | CLLS | CLLS-R | *p* | CLLS | CLLS-R | *p* |
| PUT | 0.77 | 0.72 | <0.05 | 0.67 | 0.70 | <0.05 |
| GP | 0.88 | 0.89 | 0.12 | 1.09 | 1.13 | <0.05 |
| CC | 0.57 | 0.58 | <0.05 | 1.95 | 1.98 | 0.14 |
| IC | 0.70 | 0.71 | <0.05 | 1.88 | 1.94 | <0.05 |
| EC | 0.73 | 0.72 | <0.05 | 1.12 | 1.14 | <0.05 |
| Cg | 0.92 | 0.92 | 0.70 | 1.55 | 1.51 | 0.06 |
|  | MK | | | *χ^2^* (x 10^-2^) | | |
|  | CLLS | CLLS-R | *p* | CLLS | CLLS-R | *p* |
| PUT | 0.69 | 0.70 | 0.39 | 0.93 | 0.36 | <0.05 |
| GP | 1.00 | 1.03 | <0.05 | 2.46 | 1.30 | <0.05 |
| CC | 1.09 | 1.09 | <0.05 | 2.75 | 1.88 | <0.05 |
| IC | 1.25 | 1.27 | <0.05 | 1.24 | 0.70 | <0.05 |
| EC | 0.87 | 0.88 | <0.05 | 0.80 | 0.41 | <0.05 |
| Cg | 0.97 | 0.99 | <0.05 | 1.02 | 0.64 | <0.05 |

**Abbreviations:** PUT = putamen; GP = globus pallidus; CC = corpus callosum; IC = internal capsule; EC = external capsule; Cg = cingulum.
